# Diffusion Parameters Analysis in a Content-Based Image Retrieval Task for Mobile Vision

**DOI:** 10.3390/s20164449

**Published:** 2020-08-09

**Authors:** Federico Magliani, Laura Sani, Stefano Cagnoni, Andrea Prati

**Affiliations:** 1 IMP Lab—Department of Architecture and Engineering, University of Parma, Parco Area delle Scienze 181/A, 43124 Parma, Italy; andrea.prati@unipr.it; 2 IBIS Lab—Department of Architecture and Engineering, University of Parma, Parco Area delle Scienze 181/A, 43124 Parma, Italy; laura.sani@unipr.it (L.S.); stefano.cagnoni@unipr.it (S.C.)

**Keywords:** content-based image retrieval, diffusion on graphs, genetic algorithms

## Abstract

Most recent computer vision tasks take into account the distribution of image features to obtain more powerful models and better performance. One of the most commonly used techniques to this purpose is the diffusion algorithm, which fuses manifold data and k-Nearest Neighbors (kNN) graphs. In this paper, we describe how we optimized diffusion in an image retrieval task aimed at mobile vision applications, in order to obtain a good trade-off between computation load and performance. From a computational efficiency viewpoint, the high complexity of the exhaustive creation of a full kNN graph for a large database renders such a process unfeasible on mobile devices. From a retrieval performance viewpoint, the diffusion parameters are strongly task-dependent and affect significantly the algorithm performance. In the method we describe herein, we tackle the first issue by using approximate algorithms in building the kNN tree. The main contribution of this work is the optimization of diffusion parameters using a genetic algorithm (GA), which allows us to guarantee high retrieval performance in spite of such a simplification. The results we have obtained confirm that the global search for the optimal diffusion parameters performed by a genetic algorithm is equivalent to a massive analysis of the diffusion parameter space for which an exhaustive search would be totally unfeasible. We show that even a grid search could often be less efficient (and effective) than the GA, i.e., that the genetic algorithm most often produces better diffusion settings when equal computing resources are available to the two approaches. Our method has been tested on several publicly-available datasets: Oxford5k, ROxford5k, Paris6k, RParis6k, and Oxford105k, and compared to other mainstream approaches.

## 1. Introduction

Although Content-Based Image Retrieval (CBIR) has been a relevant research topic for a long time, it is far from being a completely solved problem. The final objective is to retrieve the images belonging to a given dataset which are most similar to a query image. A common approach to this task consists of calculating global descriptors to represent images. Then, retrieval is based on the computation of the Euclidean distance between all possible image pairs to find the images which are closest to the query.

Although the problem seems simple to solve, it poses several challenges, represented, for instance, by images with different resolutions, illumination conditions, viewpoints, and so on. Additionally, the presence of distractors or background objects such as cars, people, and trees may also make it difficult for the algorithm to retrieve the right pictures [1]. These challenges are even more critical in case of mobile, distributed applications, where multiple cameras (or mobile devices, possibly carried by tourists or passers-by) can acquire different images of the scene, in terms of illumination, viewpoint, scale, etc. In other words, the development of a CBIR system capable of overcoming these challenges can open a wide variety of applications, from electronic tourist guides, to fine-grained geo-localization, to augmented reality, and so on.

Computers cannot rely on knowledge and experience as humans do, so they can not easily retrieve the right images. This problem is known as the "semantic gap" problem because a gap exists between low-level image pixel representation and high-level semantic concepts that can be derived from them [2].

The requirements for a retrieval technique are precision and speed, i.e., such techniques must retrieve the images that are closest to the query in the shortest possible time. Nevertheless, it is not always possible to achieve both goals, for several reasons related to hardware, specific dataset challenges, time constraints, etc. Therefore, the only possible final target is finding a good trade-off between the two requirements.

The introduction of deep learning and Convolutional Neural Networks (CNNs) has allowed researchers to boost retrieval results. Firstly, they have proposed to encode images using the features extracted by an intermediate layer of a CNN, trained on a huge dataset, such as ImageNet that contained images from several classes (i.e., cars, dogs, chairs, motorcycles, cats, etc.). This strategy, known as transfer learning, works well because CNNs training on such large, general-purpose datasets makes it possible for the network to learn generic features from the huge variety of images and image classes they contain. Additionally, several papers have considered the application of embedding strategies on the feature maps extracted from the CNN (the most frequently used being, at present, VGG16 and ResNet101) in order to create a global descriptor. In fact, recently, several strategies have been proposed that are aimed at making the descriptors less sensitive to scale changes, rotation, occlusions, and so on [3,4,5,6].

In doing so, they have trained CNNs with a metric-learning objective that creates better global descriptors, exploiting such embeddings. This approach requires more computational resources because it needs to re-train or fine-tune the CNN on a dataset similar to the one to which the application is aimed. From this viewpoint, different types of loss function have been proposed: triplet loss [7], contrastive loss [8], and listwise loss [9].

In addition, the advent of manifold representation and graph-based techniques as diffusion approaches have affected several computer vision tasks, such as Content-Based Image Retrieval. On this task, Iscen et al. [10] and Yang et al. [11] obtained excellent performances on several publicly available image retrieval datasets through the application of the diffusion process to R-MAC descriptors [12]. A possible reason for the success of diffusion for image retrieval [13] is its ability to identify more neighbors of the query using a manifold representation than using a Euclidean metric. In fact, the latter hypothesizes that the query neighbors lie within a sphere whose radius determines the number of the resulting images. However, if the distribution of the database elements, as usually happens, does not follow a spherically symmetrical distribution, not all possible correct results can be obtained, making it necessary to hypothesize a more complex distribution for the query neighbors, which has to be derived from the distribution of the database embeddings.

To do so, it is necessary to create the k-Nearest Neighbors (kNN) graph of the database embeddings, but this task is computationally very demanding. To solve this issue, in [14], we proposed a technique based on a pipeline for efficient and effective diffusion-based retrieval which relies on an approximate kNN graph. Briefly, the graph is constructed following a divide-and-conquer method, based on unsupervised hashing functions. Even if it needs to be repeated many times, such a method is still faster than a brute-force approach to create the kNN graph because not all the elements need to be connected.

Using this approximate kNN graph suited for diffusion, it is possible to obtain the same retrieval performance as obtained using the exact graph in a much shorter computational time.

Diffusion exploits the kNN graph and, starting from the query embedding, executes some random walks on it. This means that the next node visited will be chosen randomly among the ones directly connected to the current one. This process is repeated many times to find the best path, finding the images that are most closely related to the query, based on the resulting weights between nodes. The sequence of nodes traversed while moving along the best path corresponds to ranking the query neighbors.

Unfortunately, the diffusion process is driven by several parameters whose setting can strongly affect the performance of the retrieval task. Examples of these parameters are the number of walks to execute, the number of neighbors to detect in the graph, and the number of dataset images to consider for the random walk process. Usually, the values of these parameters are configured based on the user’s personal knowledge and on some empirical criteria or experiments. Since the optimal parameters are strongly task-dependent, one may consider designing fast search procedures to be performed as a pre-processing phase when applying diffusion to a new image retrieval task.

In this paper, we use genetic algorithms to find the best configuration of the diffusion parameters for a CBIR task, tested on different publicly available image datasets. Setting the correct values of the parameters allows one to obtain a significant extra boost of retrieval results without requiring extra effort to modify the feature extraction or embedding strategies.

Even if it is not possible to find a common configuration of the diffusion parameters that achieves the best performance on all the datasets, the best configurations are often not too different from one another. Thus, solutions found for a similar, but not the same, task can be used as initial guesses to speed up convergence to an optimal configuration. This makes GA-based optimization an effective pre-processing phase which can characterize an adaptive approach to the use of diffusion in image retrieval tasks.

The main contributions of this paper are:

- the use of genetic algorithms for tuning the diffusion parameters;
- improved efficiency of the parameter search, thanks to the intrinsic parallel nature of the proposed solution, which is easily implemented as parallel code, and to a smart termination condition;
- the comparison with other state-of-the-art optimization algorithms on several publicly-available image datasets.

The paper is structured as follows. Section 2 introduces the state of the art of parameter optimization techniques. Section 3 describes the proposed approach introducing kNN-graphs, the diffusion process, and the optimization of diffusion parameters using a GA. Section 4 reports the experimental results obtained on three publicly available datasets: Oxford5k, ROxford5k, Paris6k, RParis6k, and Oxford105k. Finally, some concluding remarks are reported.

## 2. Related Works

Several computer vision algorithms depend on parameter settings that are often hand-tuned for each particular dataset. Finding an optimal parameter configuration corresponds to a complex search problem, aimed at maximizing the quality of the algorithm according to some specific performance metrics. Even if such a configuration is often presented as being incidental to the algorithm, correctly setting these parameters is frequently critical for exploiting the full potential of a method [15], while being a hard problem because of the relatively large number of parameters that need to be tuned, their ranges, and the complex interactions that may exist between them. Configuring the parameters independently may therefore lead to sub-optimal results, whereas trying all possible combinations is often unfeasible due to the curse of dimensionality, especially considering that some parameters, to which the algorithm performance may be very sensitive, may also be real numbers.

Parameter optimization algorithms can be grouped into two main classes:

- *Parameter tuning*: the optimal parameter values are selected offline and then the algorithm is run using those values, which do not change anymore during execution [16];
- *Parameter control*: the parameter values may be adapted at runtime, according to a strategy that depends on the results that are being achieved [17,18].

Parameter tuning is the case of interest for this paper and its importance has been frequently addressed in the recent past [19,20]. Several algorithms for parameter tuning have been proposed [21,22,23], among which the simplest strategies are grid search and random search. In [24], the authors compare the performance of neural networks whose hyper-parameters have been configured using grid search and random search. Random search is shown to be more efficient than grid search and can find models that are as good or better, requiring much less computational time. Random search results are better especially when only few hyperparameters affect the final performance of the machine learning algorithm. In this case, grid search allocates too many trials to the exploration of dimensions that do not matter, suffering from poor coverage of dimensions that are important. Many other hyperparameter optimization algorithms for neural networks have been proposed recently [25,26].

When the search space is non-continuous, high-dimensional, non-convex or multi-modal, local search methods are consistently outperformed by stochastic optimization algorithms [27]. Metaheuristics are general-purpose stochastic procedures designed to solve complex optimization problems [28,29]. These optimization algorithms are non-deterministic and approximate, i.e., they do not always guarantee that they find the optimal solution, but they can find a good one in reasonable time. Metaheuristics require no particular knowledge about the problem structure other than the objective function itself, when defined, or a sampling of it. They are simple, easy to implement, and flexible enough to deal with a wide range of optimization problems in which more classical approaches fail or struggle [30,31]. The main objective of metaheuristics is to achieve a trade-off between diversification (exploration) and intensification (exploitation). Diversification implies generating diverse solutions to explore the search space on a global scale, while exploitation implies focusing the search onto a local region where good solutions have been found. An overview of the main proofs of convergence of metaheuristics to optimal solutions is presented in [32].

These optimization algorithms include:

- *Population-based methods*, in which the search process can be seen as the evolution in (discrete) time of a set of points (population of solutions) in the solution space (e.g., evolutionary algorithms [33] and particle swarm optimization [34]);
- *Trajectory methods*, in which the search process describes a trajectory in the search space and can be seen as the evolution in (discrete) time of a discrete dynamical system (e.g., simulated annealing [35] and tabu search [36]);
- *Memetic algorithms*, which are hybrid global/local search methods in which a local improvement procedure is combined with a population-based algorithm (e.g., scatter search [37]).

Evolutionary algorithms (EAs), in particular, have been very successful in solving hard, multi-modal, multi-dimensional problems in many different tasks (e.g., parameter tuning [38]). Evolutionary computing takes inspiration from biological evolution to guide the search, allowing one to perform an efficient directed search even when the dimension of the search space is large [39].

In [40], the authors present an experimental comparison of evolutionary algorithms and random search algorithms to solve the problem of the optimal control of mobile robots, showing that evolutionary algorithms can find better solutions with the same number of fitness function calculations.

Genetic algorithms (GAs) are evolutionary algorithms inspired by the process of natural selection (survival of the fittest, crossover, mutation, etc.) [41] commonly used to solve optimization problems and parameter tuning tasks. A canonical GA can be described as an algorithm that operates on solution encodings, turning a population of candidate encodings into another using a number of stochastic operators that mimick the natural principles which favor evolution. GAs and, more in general, EAs have often been used for parameter tuning in their most obvious role of quality function optimizers of direct solutions to complex design problems, described by many variables with unknown dependencies among their parameters. However, they have also been used as metaoptimizers to tune either general methods, applicable in different contexts (as, for example, the design of neural networks or classifiers [42,43,44]) or even to tune other EAs [45].

In this paper, we use a genetic algorithm to optimize the diffusion process applied to image retrieval. The diffusion parameter tuning described in this paper extends the work we presented in [46], where only preliminary tests were reported. We include many more details on the proposed solution and describe the results of an extensive experimentation on several public datasets for CBIR by which we could demonstrate that the introduction of diffusion and the optimization of its parameters significantly improve retrieval quality.

## 3. The Overall Architecture

**Figure 1:**
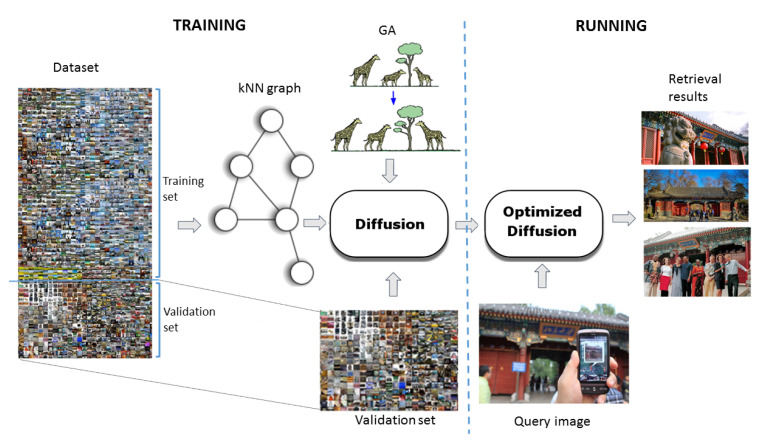
The pipeline of our optimized image retrieval system. In the training phase, after creating the approximate kNN graph, the diffusion parameters are optimized using a genetic algorithm, whose fitness is the mean average precision achieved on a “validation” subset of the image set. In running mode, our system’s retrieval quality is enhanced by the GA-optimized diffusion process.

Figure 1 describes the pipeline of the optimized diffusion-enhanced image retrieval system we propose in this paper. As for any CBIR problem, the first preliminary step consists in extracting features from the training images to compute a global descriptor for each of them. The literature on this topic has taken into consideration several techniques that improve the retrieval results by choosing descriptors that are robust to different transformations (e.g., scale, rotation, …).

Recently, as in many other fields, approaches based on deep neural networks have been particularly successful. In our system, we adopted the R-MAC descriptors [6], which are extracted by the convolutional layers of a ResNet-101 deep network, for the excellent performance exhibited on several public image datasets [10]. Using such a representation, a kNN graph is created, based on which images can be ranked according to a query. To reduce the time required by this process, we built an approximate kNN graph instead of using an exhaustive approach to create a full lossless graph. As demonstrated in [46] and, more extensively, in Section 4, the introduction of the diffusion process can produce significant improvements of retrieval quality. However, the performance of diffusion is sensitive to the setting of its parameters. This is why we applied a genetic algorithm (see Section 3.3) to optimize such parameters as a last refinement of the preliminary “training” phase.

In the following subsections, we describe the main components of our approach: the kNN graph, the diffusion process, and the genetic algorithm.

### 3.1. Approximate kNN Graph

Graphs are used in many different computer vision tasks, the most recent applications being related to: image retrieval [10], unsupervised representation learning [47], adversarial training [48], unsupervised fine-tuning of algorithms [49], semi-supervised learning [50], reinforcement learning [51], generative models [52], and classification [53].

In our case, the graph used is the k-Nearest Neighbor (kNN) graph, which is undirected and weighted. It means that the nodes, each of which represents an image in the dataset, are connected in an undirected way, i.e., the direction along which the graph is traversed from one node to its neighbor does not matter, while the weight associated with the connections expresses the similarity degree between the connected images: the larger the weight, the more similar the two images.

Let us consider an image dataset $D=\{d_{1},\cdots ,d_{N}\}$, composed of *N* images and a similarity measure θ:D×D→R. The value of the edges between nodes *i* and *j* in the graph is given by the similarity measure $\theta \left(d_{i},d_{j}\right)=\theta \left(d_{j},d_{i}\right)$. In our case, the similarity metric we adopted is the cosine similarity that can be easily calculated as the dot product of the image descriptors.

The creation of the kNN graph, a required first step in diffusion applications (see Figure 1), requires a very long time if an exhaustive, brute-force approach is used, which connects each image with all others. The method we used to build the graph is the one proposed in [14], called a Locality Sensitive Hashing (LSH) kNN graph that follows a divide-and-conquer strategy. The LSH [54] can distribute the points belonging to a dataset almost uniformly into several buckets using a hashing mechanism. The main advantage of this approach is its capability to project points that are close to each other into the same bucket with high probability.

Averaging over several repetitions of the hashing process makes it possible to obtain an effective distribution. The usual function used for hashing purposes is an isotropic Gaussian distribution N(0,I). The LSH kNN graph method reduces the time needed to create the graph, maintaining or, in some cases, improving the final retrieval performance obtained after applying the diffusion algorithm.

### 3.2. Diffusion

Following the pipeline shown in Figure 1, the next step after the kNN graph creation is the application of the diffusion process to a subset of images to optimize its parameters using a genetic algorithm.

Diffusion is a ranking method of the elements of a graph with respect to their similarity to a query, based on a random walk process. Typically, based on the kNN graph, the diffusion mechanism computes an optimal path starting from the point representing the query, along which the closest neighbors of the query (i.e., its most similar images) are visited first. The optimal path is obtained by repeatedly applying random walks: in each step, a random node, connected to the last node reached, is chosen and added to a list representing the path. This process is iterated until the best path is found, i.e., the one that maximizes the ranking function.

The function $f=\left(f_{i}\right)\in R^{N}$, N being the number of nodes of the graph, assigns a ranking score $f_{i}$ to each image $x_{i}$:

(1) $$f_{i}\left(t\right)=\sigma Sf_{i}\left(t-1\right)+\left(1-\sigma \right)y$$

where y=1 for the query and 0 for the images in the dataset, σ is a random value in the interval [0,1] and *S* identifies the affinity matrix created on the dataset elements connected in the kNN graph [13]. The values for $f_{i}\left(0\right)$ are computed as the Euclidean distance between the dataset image descriptors and the query.

**Figure 2:**
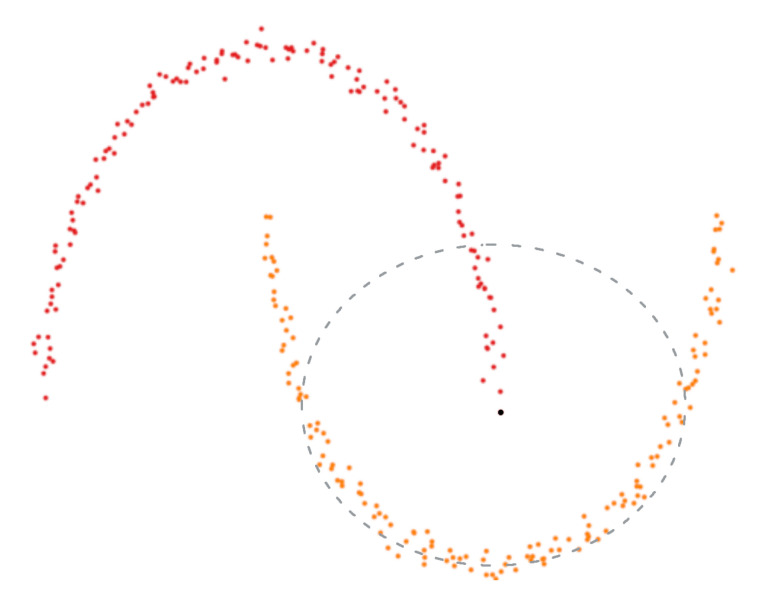
2D representation of two data distributions (red and orange dots). The black dot identifies the query and the gray circle represents the neighbors found within a range equal to its radius based on the application of the Euclidean distance. Such a metric cannot achieve an optimal performance since, as the radius of the circle is increased to include all orange dots, it soon also starts collecting false positives (red dots). The application of diffusion, which exploits the manifold distribution, achieves much better results—best viewed in color.

The diffusion mechanism applied to image retrieval is capable of yielding good final performance because it exploits the manifold distribution of the data. Figure 2 shows an example of a possible problematic case. It reports two data distributions (in 2D for the sake of simplicity) where the Euclidean metric would fail. In fact, considering the black dot as the query, the Euclidean distance (represented by a circle of variable radius in 2D) may either select too few neighbors (if the radius is small) or (if, like in the figure, the radius is large) also include points/data belonging to the other distribution, i.e., false positives, represented by red points. Instead, the diffusion mechanism takes into consideration the structure of the data within the manifold using the kNN graph. Thus, being based only on the connections between the data points, it avoids the need to make assumptions about the statistical distribution of data.

#### Diffusion Parameters

The diffusion process is regulated by several parameters, whose value can affect the final retrieval performance. In the following, we describe them and their role in the algorithm.

Experimental results from the literature demonstrate that diffusion performs better when the affinity matrix *S* is modified as follows: S=S−α*S, where α∈[0,1] represents a factor used to adjust the connections between nodes. In addition to this, other improvements can be introduced. For instance, the power iteration method [55] used for the resolution of the Page Rank problem raises to the power of β the elements of the sparse affinity matrix ($s_{ij}=s_{ij}^{\beta }$, with $s_{ij}\in S$) in order to remove useless connections in the graph. Moreover, the same principle can also be applied (with a factor γ) to the query vector $y_{i}=y_{i}^{\gamma }$, where $y_{i}\in y$.

The other diffusion parameters that need to be set are:

- $k_{s}$, the length of the random walks on the graph;
- *k*, the number of query neighbors returned in the retrieval task;
- iterations, the maximum number of iterations allowed;
- trunc, the number of images taken into consideration during the application of diffusion.

### 3.3. Genetic Algorithms for Diffusion Parameters Optimization

The next step in our pipeline, the last of what can be termed the “training phase”, is the optimization of the diffusion parameters. Optimization algorithms are characterized by the strategy according to which they explore the domain over which the target function is defined. They need to satisfy criteria that find a good trade-off between search efficiency, in terms of computational time, and its final effectiveness.

In this section, we describe how we used a genetic algorithm to optimize the diffusion parameters, with a particular focus on the choice of the termination criterion, on its impact on the results of the optimization, and on the computational time needed for the parameter tuning phase.

In the GA, we have used for tuning the diffusion parameters, each individual, corresponding to a specific configuration, is represented by a vector of seven values, corresponding to the seven parameters to be optimized. The value of the real parameter α has been set in the following range: α∈[0,1](float). The other parameters take integer values from the following sets: β∈{1,…,10}(int), γ∈{1,…,10}(int), $k_{s}\in \left\{20,\ldots ,100\right\}\;\left(int\right)$, k∈{5,…,40}(int), iterations∈{10,…,30}(int), trunc∈{3000,…,datasetsize}(int), where {a,…,b} represents the set of integer numbers {x∈Z:a≤x≤b}. These ranges have been selected based on past experiments and general knowledge of the diffusion mechanism. In order to be reasonably sure that these ranges include all plausible values, we kept them as large as possible.

The fitness function to be maximized corresponds to the mean Average Precision (mAP) obtained by the diffusion process in the retrieval phase. It is worth emphasizing that the best parameters are computed using only the validation set (see Figure 1), while keeping them unchanged during the testing phase.

The initial population, of size Pop, is obtained by generating random individuals satisfying the constraints on the parameter ranges. During the selection operation, each individual is replaced by the best of three individuals extracted randomly from the current generation (tournament selection). The selected individuals are mated with a probability CxPb, generating new individuals by means of single-point crossover. An individual is mutated with a probability MutPb, while each gene is mutated with a probability IndPb. The population is entirely replaced by the offspring (generational GA).

The termination condition of the GA strongly affects its performance and computational time. In this paper, we analyze the impact of different conditions [56], corresponding either to limiting the time (number of iterations) allowed for the algorithm to reach convergence, or to metrics that measure the degree of convergence reached by the population.

In particular:

- **Maximum number of generations**: the evolutionary process is iterated for Gen generations;
- **N-best fitness value**: the evolutionary process is stopped if there is no improvement in the best fitness value for *N* consecutive iterations;
- **Standard Deviation**: the evolutionary process is stopped when the standard deviation of the fitness values of the current generation is equal to or less than a given threshold $\epsilon_{std}\geq 0$;
- **Running Mean**: the evolutionary process is stopped when the difference between the best fitness value of the current generation and the average of the best fitness values of the last $t_{last}$ generations is equal to or less than a given threshold $\epsilon_{avg}\geq 0$;
- **Best-Worst**: the evolutionary process is stopped when the difference between the best and the worst fitness value of the current generation is equal to or less than a given threshold $\epsilon_{b-w}\geq 0$.

A buffer has been introduced to store the best individuals (the ones that produce the largest mAP) found during the evolutionary process, and their corresponding fitness (mAP) values. Thus, at the end of the run, the best parameter setting can be found not only among the individuals belonging to the population generated in the last iteration, but also among the best ones found during the whole evolutionary process, which are stored in the buffer.

## 4. Experimental Results

In this section, we illustrate the experimental results we have obtained applying our method to five publicly available datasets (Oxford5k, ROxford5k, Paris6k, RParis6k and Oxford105k) which will be described in detail in Section 4.1.

In all five experiments, we have chosen to consider the Mean Average Precision (mAP) to measure retrieval accuracy. The results of GA optimization have been compared to the results obtained by other techniques that are commonly used for parameter tuning. The genetic algorithm has been implemented using DEAP (https://deap.readthedocs.io/en/master/) (Distributed Evolutionary Algorithms in Python) [57], an evolutionary computation framework for rapid prototyping and testing.

The hardware platform on which the experiments were run featured an Intel Core i7 @ 3.40 GHz × 8 CPU equipped with 32 GB DDR4 RAM.

### 4.1. Datasets

The impact on the retrieval performance of the optimization of the diffusion parameters has been evaluated on the following publicly available CBIR image datasets:

- **Oxford5k** [58] contains 5063 images belonging to 11 classes.
- **ROxford5k** [59] contains 4993 images and represents the revisited version of the previous one. It is composed by 70 queries that are new images added to the old dataset. All images are labeled in order to test retrieval systems at three different levels of difficulty: *Easy*, *Medium*, and *Hard*;
- **Paris6k** [60] contains 6412 images belonging to 12 classes.
- Similarly to **ROxford5k**, **RParis6k** [59] contains 6322 images and represents the revisited version of the previous one. It is also composed of 70 queries that are new images added to the old dataset. All images are labelled in order to test the pipeline at three different levels of retrieval difficulty: *Easy*, *Medium*, and *Hard*;
- **Oxford105k** has been created adding the first 100 k images of **Flickr1M** to the Oxford5k dataset. **Flickr1M** [61] contains 1 million Flickr images used for large-scale evaluation. The images are divided into multiple classes and have not been specifically selected for an image retrieval task.

### 4.2. The Importance of Diffusion for Retrieval

Before reporting the results obtained by our system, we motivate our choice of applying diffusion to the kNN-graph and the resulting need for an efficient and effective algorithm to find an optimal setting for the diffusion parameters. To do so, we anticipate the global results obtained on Oxford5k and Paris6k. In the following subsections, we give many more details about the partial results which led to this final global outcome.

**Figure 3:**
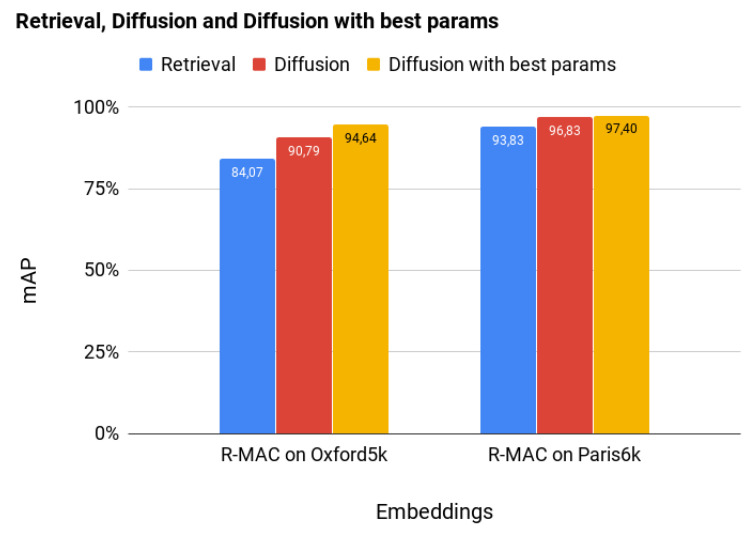
Comparison of results obtained using R-MAC descriptors [10] tested with different methodologies on Oxford5k, Paris6k—best viewed in color.

Figure 3 shows the results of the experiments on Oxford5k and Paris6k. All the methods adopt the same global descriptors (R-MAC), but the retrieval process is different. In the first case, we based retrieval only on a nearest-neighbor approach based on the Euclidean distance between the global descriptor of the query and all the images in the dataset. In the second case, we used diffusion with “standard” parameters manually selected based on previous experience. Finally, in the last case, we applied diffusion after using a GA to optimize its parameters.

Figure 3 demonstrates clearly that the adoption of diffusion can significantly improve retrieval accuracy on both datasets and that the optimized setting of the diffusion parameters can further improve it. It is worth noticing that the introduction of the GA-based optimization of the diffusion parameters requires a preliminary analysis (a so-called “meta-optimization”) of the five parameters that regulate the behaviour of the GA: Population size, Number of Iterations, Crossover Probability, Mutation Probability, and Mutation Probability for each individual gene. This initial overhead is not only aimed at letting the retrieval system achieve the best possible final performance, but also and, more importantly, at finding a “standard” setting for the GA that can both directly guarantee good performance, and, being a good “starting guess”, minimize the fine-tuning time when transferred to other benchmarks. Our expectation, finally confirmed by the results achieved, was supported by the literature on meta-optimization, which shows that the dependence of the performance of an optimizer on the parameters of a meta-optimizer is less critical than the dependence of the final outcome of the optimization on the parameters of the optimizer [45]. If this sort of “vanishing gradient” effect did not occur as the level of meta-optimization increased, i.e., if tuning a meta-optimizer was as a critical problem as tuning the optimizer, the literature on meta-optimization would not be as extended as it actually is.

Based on this consideration, we then started our experiments from the smallest of the four datasets, Oxford5k, which was firstly used to tune the parameters of the GA.

### 4.3. Results on Oxford5k

To avoid the burden of operating a full grid search on the parameters of the GA, which would dramatically reduce the efficiency of our approach, we followed a greedy exploration of the space of the GA parameters, proceeding in what our experience suggested could be a reasonable order of relevance. This means we first set sensible “average” initial values for the other parameters and studied the dependence of the results on the number of generations (see Table 1); we then set the number of generations as the one providing the best results in the previous test and tuned population size (see Table 2) keeping the other parameters constant, and so on for the following parameters (Table 3, Table 4 and Table 5).

The initial configuration of the GA was: Pop = 50, CxPb=0.5, MutPb=0.2, IndPb=0.1. The number of generations and the population size have been set to different values in the range {10, …, 100} *(int)*, considering a potential maximum budget of 5000 fitness computations. As shown in Table 1 and Table 2, the best configurations correspond to the largest numbers of fitness computations (Gen=50, Pop=50 and Gen=100, Pop=50). Since these configurations lead to the same mAP (94.40%), the other parameters of the GA have been modified starting from the configuration that requires the lowest computation load (Gen=50, Pop=50).

Table 3 shows that the retrieval accuracy reaches its best value for a crossover probability (CxPb) equal to 0.3 (94.41%). Instead, for the mutation probability (MutPb), the best results have been achieved with values 0.2 (94.41%) and 0.3 (94.41%), as shown in Table 4. Considering the mutation probability for each gene (IndPb), the highest mAP has been achieved with a value of 0.1 (94.41%).

Therefore, as shown in Table 5, the best set of parameters for the genetic algorithm thus obtained is: Gen=50, Pop=50, CxPb=0.3, MutPb=0.2, IndPb=0.1. The corresponding configuration obtained for the diffusion parameters is: α=0.97, β=3, γ=2, $k_{s}=53$, k=9, iterations=10, trunc=4136.

Finally, since, on the one hand, we had obtained the same results as with 50 individuals and, on the other hand, running a larger number of iterations offers better chances to converge to the optimal solution, we also tested the final configuration changing the number of iterations to 100 (Pop=50, CxPb=0.3, MutPb=0.2 and IndPb=0.1), obtaining a further slight improvement (mAP=94.44%). The corresponding configuration obtained for the diffusion parameters is: α=0.97, β=3, γ=1, $k_{s}=95$, k=7, iterations=10, trunc=3046.

Given the stochastic nature of the GA, five independent runs of the algorithm have been executed to assess the repeatability of the results, obtaining an average mAP of 94.39%. The results we obtained were very similar in all the runs, with a standard deviation very close to 0 (0.038). The maximum mAP we obtained was equal to 94.44% while the minimum was equal to 94.34%. These results suggested there was no need to extend the number of runs to validate the procedure more robustly from a statistical viewpoint, considering also that the final results seem to be not too sensitive to small changes in the parameter settings. Instead, we have run 30 independent repetitions of the final optimization step to evaluate the statistical significance of the results we describe in the following.

Table 6 reports the results of different optimization techniques applied to image retrieval based on an approximate kNN graph and diffusion. For each method, the table shows the mAP of the best configuration found and the corresponding computational time needed for the optimization. In order to fairly compare the methods, all the experiments have been allowed to perform the same number of fitness evaluations.

Random search [24], which is a global search technique, has sampled, in this case, 5 k or 10 k configurations modifying all the parameters according to a uniform statistical distribution. We also performed a grid search on 5 k or 10 k different parameter settings in which the values of the configurations were sampled according to a grid with pre-set step values. With the term “Manual configuration”, we refer to a setting of the diffusion parameters taken from the literature [10] without subjecting it to any further optimization method. It represents the baseline solution for the problem and it is also the one which obtains the worst final results.

A metaheuristic such as a GA which, in a sufficiently long time, can converge to any possible combination of values within its search space is, in principle, preferable to a grid search. The latter is constrained to exploring a pre-set number of possible solutions, a large part of which would generally lie in poorly performing regions of the diffusion parameter space, which a smarter search would rule out after few evaluations. On the other hand, as we showed above, a GA needs some parameter tuning to perform at its best and, even after tuning, it cannot guarantee that, within a given time or fitness evaluation budget, its result will be better than those of a similarly computationally demanding grid search. However, if we observe the computational time reported in the tables for a grid search and a GA with equal budget in terms of number of settings to test, we observe that the GA converges to its best solution in a much shorter time. In fact, since the complexity of evaluating a parameter set is virtually the same whatever the context, the shorter time taken by the GA to converge indicates that it reaches a termination condition before using the whole budget. In addition, when the GA is close to convergence, it usually happens that some individuals in the population are identical. In that case, their fitness is evaluated only once for all of them, which also reduces convergence time.

This makes the complexity of a grid search comparable to testing a few settings of the GA, which, as we will see in the end, would be probably enough to find the optimal setting. A parallel version of the GA, distributed over five parallel processes during fitness evaluation, achieves the same result as the single-CPU GA execution in a much shorter time (6295 s).

In terms of model complexity, considering the minimum relative computation load that the application of genetic operators require with respect to a fitness evaluation, a complexity of O($N_{ev}$) can be attributed to a GA, where $N_{ev}$ is the number of fitness evaluations. In terms of intrinsic memory allocation, a GA only needs to memorize the population and some punctual statistics, which is negligible with respect to the memory occupied by the data. We have compared the methods we have taken into consideration by assigning to each of them the same maximum budget in terms of fitness evaluations. In this regard, one can observe that DEAP checks the diversity (number of different individuals) of the population and computes only once the fitness of equal individuals that tend to be more and more as the population converges towards the optimum. This fact allows the GA to perform fewer fitness evaluations than the budget even when the MaxGen termination condition is chosen. The application of the termination conditions further reduces the number of fitness evaluations causing only a marginal performance decrease.

The final and most important outcome in favor of the GA is that, in all previously-reported experiments, the latter, in whatever configuration, has always performed better than manual configuration and random search and very closely to the grid search. The only exceptions are a few settings with very low values for some of the GA parameters, which were tested only to estimate a lower bound for their magnitude, below which it is actually unreasonable to go. Oddly enough, since the GA was tuned on Oxford5k, this dataset was the only one on which the best result of the GA did not clearly outperform the grid search. That happens, instead, on the other datasets, even without any further refinement of the GA settings, i.e., taking the settings computed on Oxford5k as a default configuration.

**Figure 4:**
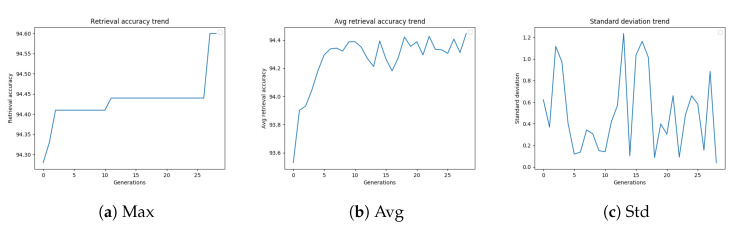
GA best retrieval accuracy, average retrieval accuracy, and standard deviation trend on Oxford5k.

As described in Section 3.3, we evaluated several stopping criteria for the GA. These termination conditions were based on the best fitness value obtained up to that generation or on the average or standard deviation of the fitness values of the population over subsequent generations. These trends are shown in Figure 4a–c, and are related to the GA configuration, which led to the best-performing settings of the diffusion parameters.

Figure 4a shows the retrieval accuracy trend of a genetic algorithm on Oxford5k. The best fitness value increases with the number of generations. As shown in Figure 4b, also the average retrieval accuracy generally tends to increase with the generations, but this trend is less stable. The standard deviation trend of the mAP values of the population, shown in Figure 4c, is probably strongly affected by the outcome of mutations, considering that the population is quite small.

Table 7 summarizes the results obtained using the GA termination conditions described in Section 3.3. The “maximum number of generations” (“Max Gen”) criterion achieves the best retrieval accuracy (94.41%), but it takes longer than the other techniques. The “Standard deviation” criterion is very fast, with a somewhat poorer performance (94.23%), while the “Best-Worst” termination condition reaches a mAP equal to 94.39% in 4206 s, which probably represents the best trade-off between execution time and solution quality. It is worth noting that the results corresponding to the different stopping criteria have been all obtained running the parallel GA implementation.

Collecting the statistics over 30 runs of the stochastic methods we take into consideration (GA with different termination conditions, random search), and performing Wilkinson’s rank sum test (*p* < 0.05), we can reinforce the conclusions reported above, which apply also to the other datasets, as follows:

- The improvement in terms of both mAP and execution time introduced by all GA-based methods with respect to a random search (Tables 6, 8, 10, 12 and 14) are always statistically significant.
- Regarding the comparison between the different termination conditions (Tables 7, 9, 11, 13 and 15), the statistical significance of the difference between the best-performing algorithm and the others is robustly correlated with the value of the difference. In the case of Oxford5k, performing the whole budget of 5000 evaluations (MaxGen) yields significantly better results than all other termination conditions (even if by a relatively small gap). Obviously, that advantage is compensated by a significantly higher time needed to run the experiments, since the other methods can stop the optimization process when a sensible condition is reached.

### 4.4. Results on ROxford5k

This section describes the results we obtained on ROxford5k dataset.

Table 8 reports the results of different tuning techniques applied to the diffusion process. For each technique, the table shows the results of the best configuration found. The results have been compared in terms of mAP, running time, and number of fitness computations. The best diffusion parameter set we found is: α=0.98, β=5, γ=3, $k_{s}=75$, k=8, iterations=12, trunc=3219.

**Figure 5:**
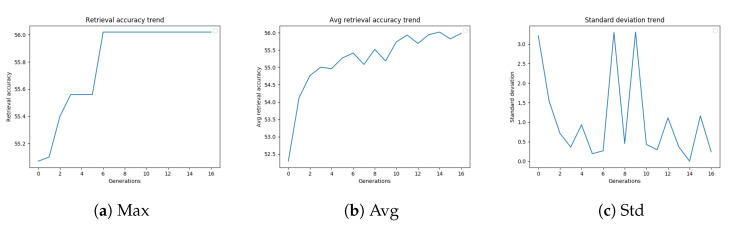
GA best retrieval accuracy, average retrieval accuracy and standard deviation trend on ROxford5k

In order to better appreciate the results obtained using different stopping criteria, Figure 5a–c display the trend of the retrieval accuracy (best, average, and standard deviation, respectively) versus the number of generations.

Table 9 summarizes the results of different termination conditions. The “Best-Worst” termination condition reached the same mAP obtained by “Max Gen” (56.56%) with a smaller number of fitness evaluations.

### 4.5. Results on Paris6k

This section describes the results we obtained on Paris6k dataset. Table 10 reports the results of different tuning methods applied to Paris6k. The best final configuration of the diffusion parameters, achieving a mAP of 97.32%, was: α=0.87, β=1, γ=2, $k_{s}=40$, k=11, iterations=10, trunc=3761. As with the previous dataset, optimizing diffusion parameters obviously takes more time than directly using “manual configuration", but it improves the final retrieval performance of the diffusion process.

**Figure 6:**
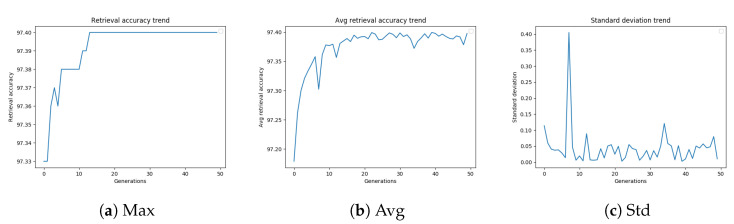
GA best retrieval accuracy, average retrieval accuracy, and standard deviation trend on Paris6k.

Figure 6a–c shows the trend of the retrieval accuracy (best, average, and standard deviation, respectively) versus the number of generations.

Table 11 reports the results of different termination conditions for GA applied for the optimization of diffusion parameters. The best retrieval accuracy has been obtained by the “N-best fitness” and “Max Gen” stopping criteria. “N-best fitness” reached the same mAP obtained by “Max Gen” (97.40%) but after executing a smaller number of fitness evaluations.

### 4.6. Results on RParis6k

Table 12 summarizes the results of different tuning techniques applied on the diffusion process. For each technique, the table shows the result of the best configuration found. The results have been compared in terms of mAP, running time, and number of fitness evaluations.

**Figure 7:**
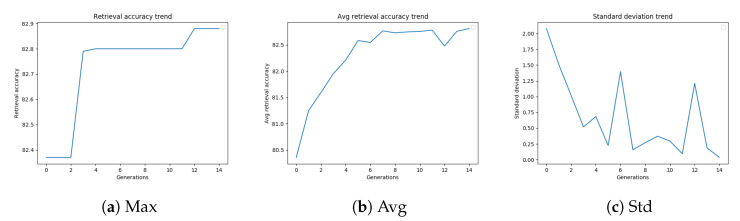
GA best retrieval accuracy, average retrieval accuracy, and standard deviation trend on RParis6k

Figure 7a–c shows the retrieval accuracy values (best, average, and standard deviation, respectively) versus the number of generations.

Table 13 summarizes the results obtained by the GA-optimized diffusion parameter settings using the different termination conditions taken into consideration. All the stopping criteria obtain the same retrieval accuracy (83.05%). The fastest criterion is “Running mean” (15,514 s), which executes 1300 fitness computations (less than half as many fitness evaluations as performed when “Max Gen” criterion is adopted).

### 4.7. Results on Oxford105k

Given the large dimension of Oxford105k dataset, the ranges of parameters $k_{s}$ and *k* have been extended to {20,…,250}(int) and {5,…,100}(int), respectively.

Table 14 reports the results of different tuning methods applied to the parameters of the diffusion technique on the Oxford105k dataset. The best final configuration of the diffusion parameters, achieving a mAP of 94.20%) was the following: α=0.97, β=2, γ=1, $k_{s}=68$, k=7, iterations=10, trunc=18353. The “manual configuration” is obviously faster than the GA, but the final performance is very different: the GA obtains 94.20% while the “manual configuration” achieves only 92.50%.

**Figure 8:**
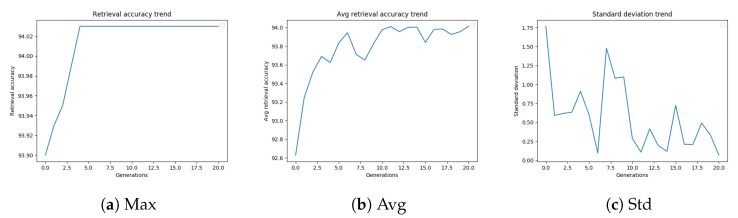
GA best retrieval accuracy, average retrieval accuracy and standard deviation trend on Oxford105k.

Figure 8a–c shows the trend of the retrieval accuracy (best, average, and standard deviation, respectively) versus the number of generations.

Table 15 reports the results obtained using different GA termination conditions. The best retrieval accuracy has been obtained by the “Max Gen” criterion.

### 4.8. Comparison with Other State-of-the-Art Diffusion Methods

Table 16 reports a comparison between our work and other state-of-the-art approaches based on the diffusion method. We adopt mean Average Precision (mAP) as the evaluation metric because it is the metric used in all the compared works for evaluating the results obtained. We compared our proposed method with other state-of-the-art approaches on three different datasets: Oxford5k, Paris6k, and Oxford105k. We have not replicated the experiments, but we just list the results reported by the authors in the papers referenced in the table.

Our work outperforms other state-of-the-art algorithms which use global descriptors [10,11] (see upper half of Table 16). In Iscen et al. [10] and Yang et al. [11], as well as in Xu et al. [62], diffusion was also applied to regional R-MAC descriptors (see lower half of Table 16). In particular, Yang et al. [11] adopts both global and regional descriptors to achieve the best performance in the last row of the table. In particular, they compute a weighted mean of scores from regional and global diffusion, setting the weight for regional diffusion to 0.75. This modification generally improves the final performance, but at the cost of a much longer computational time. Nonetheless, our approach still achieves comparable results but at much less time since diffusion on global descriptors is much faster than that on regional descriptors.

## 5. Conclusions

This paper proposes a genetic algorithm as a way of finding the best configuration of the diffusion parameters for Content-Based Image Retrieval. Using an approximate kNN graph, the diffusion process starts from the point corresponding to the query image and follows the best path in order to find the neighbors of the query, i.e., the images that are most similar to it.

The retrieval accuracy has been evaluated using the mAP obtained by the diffusion process. The GA-tuned diffusion parameters allowed us to achieve good retrieval results on several public image datasets, outperforming in most cases the results obtained using other techniques (manual configuration, random search, and grid search).

Considering possible limitations of our approach, it has to be noticed that, despite our goal of finding a common set of parameters for all the datasets, it turned out that the optimization needs to be tailored to each particular dataset. Table 17 summarizes the optimal values of the diffusion parameters found by the GA for the image datasets we took into consideration: Oxford5k, ROxford5k, Paris6k, RParis6k, and Oxford105k.

The GA-based optimization is a computationally expensive and time-consuming procedure, but it is executed only once in the retrieval pipeline. To deal with this problem, we have also implemented a parallel version of the GA optimization, dispatching the computation on five parallel processes, which reduced significantly the time required by this step. GA optimization time can be further slightly reduced by avoiding to evaluate more than once identical individuals that are often present in the final generations, with no effects on the final result. A more relevant reduction of computation time can be achieved by adopting the termination conditions described in this paper, at the expense of a very slight performance decrease.

A statistical analysis of the results, based on a fair comparison in which we allocated the same budget to both methods, in terms of number of evaluations of different configurations, indicates that GA-based optimization is always preferable to random search, being both faster and better performing than the latter. At the same time, among the possible termination strategies, MaxGen is preferable from a performance viewpoint, since it obviously guarantees the best results. Under stringent time constraint, the application of the termination conditions yields slightly worse results in a significantly shorter time. Standard deviation is faster but tends to converge prematurely and generally performs worse than the others, while there appears to be no clear indication as to which of the other termination strategies is preferable. Moreover, thanks to either the termination strategies or the fact that DEAP does not evaluate the same individual twice when more copies of it appear in the same generation, or to both features, GA-based optimization appears to be faster than a grid search with the same evaluations budget, while guaranteeing comparable, and often better, performances.

The main limitation of our method is that, theoretically speaking, adding new images would require a new tuning of diffusion parameters through the use of GA. However, our experiments demonstrate that, if the optimization is run on a sufficiently large number of images and not too many images are added, the parameters selected by GA can remain the same with no need of running the GA again. Of course, if too many (or too different) images were added, causing relevant changes in the underlying manifold of data, a new run of GA would be necessary to better catch the manifold data structure.

Another reasonably possible limitation to be considered for our method, in its practical use, could be that the GA configuration may depend on the data to which the method is applied. This would suggest that a systematic search of the optimal GA parameters should be made for every dataset to which the method is applied. A global analysis of the results obtained on the five benchmarks shows that the GA configuration is almost insensitive to the dataset under consideration. Taking the optimal setting for the GA parameters on the Oxford5k as our default allowed us to quickly find the optimal GA setting also for the other benchmarks. Table 18 summarizes the optimal values of the GA parameters used in optimizing diffusion for the image datasets we took into consideration: it is worth noticing that only crossover probability changes, assuming two possible values over the five datasets, which are not so different from each other. This shows that tuning the GA appears not to be too critical for this application and that a GA configuration which works well on a dataset is very likely to do so also on other datasets, even when it is sub-optimal. In that case, only a limited fine-tuning in the neighborhood of the ’standard’ configuration we found may be necessary to obtain the very best results.

As future work, we will further study the dependence of the GA on its parameters, with the aim of improving its effectiveness using Meta level Evolutionary Algorithms (Meta-EAs), methods that tune evolutionary algorithms to optimize their performance, and its efficency using parameter control methods which adaptively optimize EA parameters at runtime.

## Figures and Tables

**Table 1:** Values of mAP obtained on Oxford5k by a configuration optimized by GAs running different numbers of generations (Gen). Best mAP in boldface.

Gen	Pop	CxPb	MutPb	IndPb	mAP
10	50	0.5	0.2	0.1	94.31%
20	50	0.5	0.2	0.1	94.31%
50	50	0.5	0.2	0.1	**94.40%**
100	50	0.5	0.2	0.1	**94.40%**

**Table 2:** Values of mAP obtained on Oxford5k by a configuration optimized by GAs with different population sizes (Pop). Best mAP in boldface.

Gen	Pop	CxPb	MutPb	IndPb	mAP
50	10	0.5	0.2	0.1	94.32%
50	20	0.5	0.2	0.1	94.16%
50	50	0.5	0.2	0.1	**94.40%**
50	100	0.5	0.2	0.1	94.36%

**Table 3:** Values of mAP obtained on Oxford5k by a configuration optimized by GAs with different values of crossover probability (CxPb). Best mAP in boldface.

Gen	Pop	CxPb	MutPb	IndPb	mAP
50	50	0.1	0.2	0.1	93.73%
50	50	0.3	0.2	0.1	**94.41%**
50	50	0.5	0.2	0.1	94.40%
50	50	0.8	0.2	0.1	94.36%
50	50	1.0	0.2	0.1	94.34%

**Table 4:** Values of mAP obtained on Oxford5k by a configuration optimized by GAs with different values of mutation probability (MutPb). Best mAP in boldface.

Gen	Pop	CxPb	MutPb	IndPb	mAP
50	50	0.3	0.1	0.1	93.73%
50	50	0.3	0.2	0.1	**94.41%**
50	50	0.3	0.3	0.1	**94.41%**
50	50	0.3	0.4	0.1	94.32%
50	50	0.3	0.5	0.1	94.31%

**Table 5:** Values of mAP obtained on Oxford5k by a configuration optimized by GAs with different values of mutation probability for each gene (IndPb). Best mAP in boldface.

Gen	Pop	CxPb	MutPb	IndPb	mAP
50	50	0.3	0.2	0.1	**94.41%**
50	50	0.3	0.2	0.3	94.40%
50	50	0.3	0.2	0.5	94.27%
50	50	0.3	0.2	0.8	94.23%
50	50	0.3	0.2	1.0	94.22%

**Table 6:** Comparison of different approaches to the tuning of the diffusion parameters on Oxford5k in terms of mAP, computational time, and number of fitness evaluations. Best mAP in boldface.

Method	Fitness Computations	Time	mAP
Manual configuration [14]	1	2 s	90.95%
Random search [24]	5000	29,045 s	93.67%
Random search [24]	10,000	41,610 s	93.81%
Grid search	5000	28,680 s	92.73%
Grid search	10,000	42,513 s	**94.43%**
GA	5000	17,695 s	94.41%
Parallel GA	5000	6295 s	94.41%

**Table 7:** Comparison of different GA stopping criteria (Gen=100, N=20, $\epsilon_{std}=0.5$, $t_{last}=10$, $\epsilon_{avg}=0.5$ and $\epsilon_{b-w}=0.5$) on Oxford5k in terms of mAP, time and number of fitness computations. Best mAP in boldface.

Method	Fitness Computations	Time	mAP
Max Gen	5000	6295 s	**94.41%**
N-best fitness	2500	3131 s	94.37%
Standard Deviation	550	672 s	94.23%
Running mean	1550	1941 s	94.31%
Best-Worst	3350	4206 s	94.39%

**Table 8:** Comparison of different approaches to the tuning of the diffusion parameters on ROxford5k in terms of mAP, time, and number of fitness computations. Best mAP in boldface.

Method	Fitness Computations	Time	mAP
Manual configuration [14]	1	5 s	52.50%
Random search [24]	5000	96,868 s	55.39%
Random search [24]	10,000	180,123 s	55.45%
Grid search	5000	94,356 s	55.41%
Grid search	10,000	179,567 s	55.51%
GA	5000	71,923 s	**56.56%**
Parallel GA	5000	45,196 s	**56.56%**

**Table 9:** Comparison of different stopping criteria for GA optimization (Gen=100, N=20, $\epsilon_{std}=0.5$, $t_{last}=10$, $\epsilon_{avg}=0.5$ and $\epsilon_{b-w}=0.5$) on ROxford5k in terms of mAP, time and number of fitness evaluations. Best mAP in boldface.

Method	Fitness Computations	Time	mAP
Max Gen	5000	45,196 s	**56.56%**
N-best fitness	2750	24,774 s	56.35%
Standard Deviation	600	5182 s	55.94%
Running mean	1500	13,168 s	56.34%
Best-Worst	4400	39,502 s	**56.56%**

**Table 10:** Comparison of different approaches to the tuning of the diffusion parameters on Paris6k. Best mAP in boldface.

Method	Fitness Computations	Time	mAP
Manual configuration [14]	1	4 s	97.01%
Random search [24]	5000	28,916 s	97.29%
Random search [24]	10,000	58,708 s	97.25%
Grid search	5000	33,067 s	97.26%
Grid search	10,000	59,777 s	97.26%
GA	5000	18,787 s	**97.40%**
Parallel GA	5000	6420 s	**97.40%**

**Table 11:** Comparison of different stopping criteria for GA optimization (Gen=50, N=10, $\epsilon_{std}=0.5$, $t_{last}=10$, $\epsilon_{avg}=0.5$ and $\epsilon_{b-w}=0.5$) on Paris6k in terms of mAP, time, and number of fitness evaluations. Best mAP in boldface.

Method	Fitness Computations	Time	mAP
Max Gen	5000	6420 s	**97.40%**
N-best fitness	3200	4048 s	**97.40%**
Standard Deviation	450	569 s	97.33%
Running mean	1650	2067 s	97.38%
Best-Worst	600	746 s	97.33%

**Table 12:** Comparison of different approaches to the tuning of the diffusion parameters on RParis6k in terms of mAP, time and number of fitness computations. Best mAP in boldface.

Method	Fitness Computations	Time	mAP
Manual configuration [14]	1	4 s	80.80%
Random search [24]	5000	101,235 s	82.51%
Random search [24]	10,000	197,864 s	82.78%
Grid search	5000	98,743 s	82.64%
Grid search	10,000	192,412 s	82.80%
GA	5000	85,134 s	**83.05%**
Parallel GA	5000	60,480 s	**83.05%**

**Table 13:** Comparison of different stopping criteria for GA optimization (Gen=50, N=10, $\epsilon_{std}=0.5$, $t_{last}=10$, $\epsilon_{avg}=0.5$ and $\epsilon_{b-w}=0.5$) on RParis6k in terms of mAP, time, and number of fitness evaluations. Best mAP in boldface.

Method	Fitness Computations	Time	mAP
Max Gen	5000	60,480 s	**83.05%**
N-best fitness	2250	26,654 s	**83.05%**
Standard Deviation	1400	16,601 s	**83.05%**
Running mean	1300	15,514 s	**83.05%**
Best-Worst	4600	55,655 s	**83.05%**

**Table 14:** Comparison of different approaches to the tuning of the diffusion parameters on Oxford105k. Best mAP in boldface.

Method	Fitness Computations	Time	mAP
Manual configuration [14]	1	13 s	92.50%
Random search [24]	5000	90,780 s	93.65%
Random search [24]	10,000	197,501 s	93.70%
Grid search	5000	91,243 s	93.85%
Grid search	10,000	201,546 s	94.10%
GA	5000	63,911 s	**94.20%**
Parallel GA	5000	30,120 s	**94.20%**

**Table 15:** Comparison of different stopping criteria for GA optimization (Gen=100, N=20, $\epsilon_{std}=0.5$, $t_{last}=10$, $\epsilon_{avg}=0.5$ and $\epsilon_{b-w}=0.5$) on Oxford105k in terms of mAP, time, and number of fitness evaluations. Best mAP in boldface.

Method	Fitness Computations	Time	mAP
Max Gen	5000	30,120 s	**94.20%**
N-best fitness	2200	13,093 s	94.03%
Standard Deviation	1500	8804 s	94.03%
Running mean	1850	10,979 s	94.03%
Best-Worst	3850	23,152 s	94.03%

**Table 16:** Comparison with state-of-the-art diffusion methods in terms of mAP. Above: approaches based on global features. Below: approaches based on regional features.

Method	Oxford5k	Paris6k	Oxford105k
Iscen et al. (global) [10]	87.10%	96.50 %	87.40%
Yang et al. (global) [11]	92.60%	97.10%	91.80%
Our work	94.41%	97.40%	94.20%
Xu et al. [62]	92.00%	96.60%	87.20%
Iscen et al. (regional) [10]	95.80%	96.90 %	94.20%
Yang et al. (regional) [11]	95.90%	97.60%	94.80%
Yang et al. (global+regional with late fusion) [11]	96.20%	97.80%	95.20%

**Table 17:** Summary of the best diffusion parameters for each image dataset.

Parameter	Oxford5k	ROxford5k	Paris6k	RParis6k	Oxford105k
α	0.97	0.98	0.87	0.98	0.97
β	3	5	1	6	2
γ	1	3	2	1	1
$k_{s}$	95	75	40	47	68
*k*	7	8	11	5	7
iterations	10	12	10	20	10
truncation	3046	3219	3761	5080	18353

**Table 18:** Summary of the best GA parameters for each image dataset.

Parameter	Oxford5k	ROxford5k	Paris6k	RParis6k	Oxford105k
Gen	100	100	100	100	100
Pop	50	50	50	50	50
CxPb	0.3	0.3	0.5	0.5	0.5
MutPb	0.2	0.2	0.2	0.2	0.2
IndPb	0.1	0.1	0.1	0.1	0.1

## References

[1] Magliani F., Fontanini T., Prati A. (2019). Landmark Recognition: From Small-Scale to Large-Scale Retrieval. Recent Advances in Computer Vision.

[2] Hare J.S., Lewis P.H., Enser P.G., Sandom C.J. (2006). Mind the gap: Another look at the problem of the semantic gap in image retrieval. Multimedia Content Analysis, Management, and Retrieval 2006.

[3] Babenko A., Lempitsky V. Aggregating local deep features for image retrieval. Proceedings of the IEEE International Conference on Computer Vision and Pattern Recognition.

[4] Kalantidis Y., Mellina C., Osindero S. (2016). Cross-dimensional weighting for aggregated deep convolutional features. European Conference on Computer Vision.

[5] Magliani F., Prati A. An accurate retrieval through R-MAC+ descriptors for landmark recognition. Proceedings of the ACM 12th International Conference on Distributed Smart Cameras.

[6] Tolias G., Sicre R., Jégou H. (2015). Particular object retrieval with integral max-pooling of CNN activations. arXiv.

[7] Gordo A., Almazán J., Revaud J., Larlus D. (2016). Deep image retrieval: Learning global representations for image search. European Conference on Computer Vision.

[8] Radenović F., Tolias G., Chum O. (2018). Fine-tuning CNN image retrieval with no human annotation. IEEE Trans. Pattern Anal. Mach. Intell..

[9] Revaud J., Almazan J., de Rezende R.S., de Souza C.R. Learning with Average Precision: Training Image Retrieval with a Listwise Loss. Proceedings of the International Conference on Computer Vision.

[10] Iscen A., Tolias G., Avrithis Y.S., Furon T., Chum O. Efficient Diffusion on Region Manifolds: Recovering Small Objects with Compact CNN Representations. Proceedings of the IEEE Conference on Computer Vision and Pattern Recognition.

[11] Yang F., Hinami R., Matsui Y., Ly S., Satoh S. Efficient Image Retrieval via Decoupling Diffusion into Online and Offline Processing. Proceedings of the AAAI Conference on Artificial Intelligence.

[12] Gordo A., Almazan J., Revaud J., Larlus D. (2017). End-to-end learning of deep visual representations for image retrieval. Int. J. Comput. Vis..

[13] Zhou D., Weston J., Gretton A., Bousquet O., Schölkopf B. (2004). Ranking on data manifolds. Advances in Neural Information Processing Systems 16.

[14] Magliani F., McGuiness K., Mohedano E., Prati A. An Efficient Approximate kNN Graph Method for Diffusion on Image Retrieval. Proceedings of the 20th International Conference on Image Analysis and Processing.

[15] Bergstra J., Yamins D., Cox D.D. Making a science of model search: Hyperparameter optimization in hundreds of dimensions for vision architectures. Proceedings of the 30th International Conference on Machine Learning.

[16] Hoos H.H. (2011). Automated algorithm configuration and parameter tuning. Autonomous Search.

[17] Eiben Á.E., Hinterding R., Michalewicz Z. (1999). Parameter control in evolutionary algorithms. IEEE Trans. Evol. Comput..

[18] Karafotias G., Hoogendoorn M., Eiben Á.E. (2015). Parameter Control in Evolutionary Algorithms: Trends and Challenges. IEEE Trans. Evol. Comput..

[19] Montero E., Riff M.C., Rojas-Morales N. (2018). Tuners review: How crucial are set-up values to find effective parameter values?. Eng. Appl. Artif. Intell..

[20] Sipper M., Fu W., Ahuja K., Moore J.H. (2018). Investigating the parameter space of evolutionary algorithms. BioData Min..

[21] Bergstra J.S., Bardenet R., Bengio Y., Kégl B. (2011). Algorithms for hyper-parameter optimization. Advances in Neural Information Processing Systems.

[22] Falkner S., Klein A., Hutter F. (2018). Bohb: Robust and efficient hyperparameter optimization at scale. arXiv.

[23] Imbault F., Lebart K. A stochastic optimization approach for parameter tuning of support vector machines. Proceedings of the 17th International Conference on Pattern Recognition.

[24] Bergstra J., Bengio Y. (2012). Random search for hyper-parameter optimization. J. Mach. Learn. Res..

[25] Domhan T., Springenberg J.T., Hutter F. Speeding up automatic hyperparameter optimization of deep neural networks by extrapolation of learning curves. Proceedings of the Twenty-Fourth International Joint Conference on Artificial Intelligence.

[26] Maclaurin D., Duvenaud D., Adams R. Gradient-based hyperparameter optimization through reversible learning. Proceedings of the International Conference on Machine Learning.

[27] Grefenstette J.J. (1986). Optimization of control parameters for genetic algorithms. IEEE Trans. Syst. Man Cybern..

[28] Glover F.W., Kochenberger G.A. (2006). Handbook of Metaheuristics.

[29] Engelbrecht A.P. (2007). Computational Intelligence: An Introduction.

[30] Yang X.S., Cui Z., Xiao R., Gandomi A.H., Karamanoglu M. (2013). Swarm Intelligence and Bio-Inspired Computation: Theory and Applications.

[31] Mesejo P., Ibáñez O., Cordón O., Cagnoni S. (2016). A survey on image segmentation using metaheuristic-based deformable models: State of the art and critical analysis. Appl. Soft Comput..

[32] Gutjahr W.J., Maniezzo V., Stützle T., Voß S. (2010). Convergence Analysis of Metaheuristics. Matheuristics: Hybridizing Metaheuristics and Mathematical Programming.

[33] Bäck T., Schwefel H.P. (1993). An overview of evolutionary algorithms for parameter optimization. Evol. Comput..

[34] Poli R., Kennedy J., Blackwell T. (2007). Particle swarm optimization. Swarm Intell..

[35] Kirkpatrick S., Gelatt C.D., Vecchi M.P. (1983). Optimization by simulated annealing. Science.

[36] Glover F., Laguna M. (1998). Tabu search. Handbook of Combinatorial Optimization.

[37] Glover F., Laguna M., Martí R. (2003). Scatter search. Advances in Evolutionary Computing.

[38] Rasku J., Musliu N., Kärkkäinen T. (2019). On automatic algorithm configuration of vehicle routing problem solvers. J. Veh. Routing Algorithms.

[39] Eiben A.E., Smith J.E. (2015). Introduction to Evolutionary Computing.

[40] Konstantinov S., Diveev A., Balandina G., Baryshnikov A. (2019). Comparative Research of Random Search Algorithms and Evolutionary Algorithms for the Optimal Control Problem of the Mobile Robot. Procedia Comput. Sci..

[41] Goldberg D.E. (1989). Genetic Algorithms in Search, Optimization and Machine Learning.

[42] Hamdia K., Zhuang X., Rabczuk T. (2020). An efficient optimization approach for designing machine learning models based on genetic algorithm. Neural Comput. Appl..

[43] Sun Y., Xue B., Zhang M., Yen G.G. (2020). Evolving Deep Convolutional Neural Networks for Image Classification. IEEE Trans. Evol. Comput..

[44] Nalepa J., Kawulok M. (2019). Selecting training sets for support vector machines: A review. Artif. Intell. Rev..

[45] Ugolotti R., Sani L., Cagnoni S. (2019). What Can We Learn from Multi-Objective Meta-Optimization of Evolutionary Algorithms in Continuous Domains?. Mathematics.

[46] Magliani F., Sani L., Cagnoni S., Prati A. Genetic Algorithms for the Optimization of Diffusion Parameters in Content-Based Image Retrieval. Proceedings of the ACM Proceedings of the 13th International Conference on Distributed Smart Cameras.

[47] Veličković P., Fedus W., Hamilton W.L., Liò P., Bengio Y., Hjelm R.D. (2018). Deep graph infomax. arXiv.

[48] Bojchevski A., Shchur O., Zügner D., Günnemann S. (2018). Netgan: Generating graphs via random walks. arXiv.

[49] Iscen A., Tolias G., Avrithis Y., Chum O. Mining on Manifolds: Metric Learning without Labels. Proceedings of the IEEE Conference on Computer Vision and Pattern Recognition.

[50] Douze M., Szlam A., Hariharan B., Jégou H. Low-shot learning with large-scale diffusion. Proceedings of the IEEE Conference on Computer Vision and Pattern Recognition.

[51] You J., Liu B., Ying Z., Pande V., Leskovec J. (2018). Graph convolutional policy network for goal-directed molecular graph generation. Advances in Neural Information Processing Systems.

[52] You J., Ying R., Ren X., Hamilton W.L., Leskovec J. (2018). Graphrnn: Generating realistic graphs with deep auto-regressive models. arXiv.

[53] Li D., Hung W.C., Huang J.B., Wang S., Ahuja N., Yang M.H. (2016). Unsupervised visual representation learning by graph-based consistent constraints. European Conference on Computer Vision.

[54] Indyk P., Motwani R. Approximate nearest neighbors: Towards removing the curse of dimensionality. Proceedings of the Thirtieth Annual ACM Symposium on Theory of Computing.

[55] Mises R., Pollaczek-Geiringer H. (1929). Praktische Verfahren der Gleichungsauflösung. J. Appl. Math. Mech./Z. Angew. Math. Mech..

[56] Ghoreishi S.N., Clausen A., Jørgensen B.N. Termination Criteria in Evolutionary Algorithms: A Survey. Proceedings of the International Joint Conference on Computational Intelligence.

[57] Fortin F.A., Rainville F.M.D., Gardner M.A., Parizeau M., Gagné C. (2012). DEAP: Evolutionary algorithms made easy. J. Mach. Learn. Res..

[58] Philbin J., Chum O., Isard M., Sivic J., Zisserman A. Object Retrieval with Large Vocabularies and Fast Spatial Matching. Proceedings of the IEEE Conference on Computer Vision and Pattern Recognition.

[59] Radenović F., Iscen A., Tolias G., Avrithis Y., Chum O. Revisiting Oxford and Paris: Large-Scale Image Retrieval Benchmarking. Proceedings of the IEEE Conference on Computer Vision and Pattern Recognition.

[60] Philbin J., Chum O., Isard M., Sivic J., Zisserman A. Lost in quantization: Improving particular object retrieval in large scale image databases. Proceedings of the IEEE Conference on Computer Vision and Pattern Recognition.

[61] Huiskes M.J., Lew M.S. The MIR flickr retrieval evaluation. Proceedings of the 1st ACM international conference on Multimedia Information Retrieval.

[62] Xu J., Wang C., Qi C., Shi C., Xiao B. (2018). Iterative manifold embedding layer learned by incomplete data for large-scale image retrieval. IEEE Trans. Multimed..

